# Novel Sequence-Based Mapping of Recently Emerging H5NX Influenza Viruses Reveals Pandemic Vaccine Candidates

**DOI:** 10.1371/journal.pone.0160510

**Published:** 2016-08-05

**Authors:** Christopher S. Anderson, Marta L. DeDiego, Juilee Thakar, David J. Topham

**Affiliations:** 1 New York Influenza Center of Excellence, David Smith Center for Immunology and Vaccine Biology, Department of Microbiology and Immunology, University of Rochester Medical Center, Rochester, New York, United States of America; 2 Department of Biostatistics and Computational Biology, University of Rochester Medical Center, Rochester, New York, United States of America; Icahn School of Medicine at Mount Sinai, UNITED STATES

## Abstract

Recently, an avian influenza virus, H5NX subclade 2.3.4.4, emerged and spread to North America. This subclade has frequently reassorted, leading to multiple novel viruses capable of human infection. Four cases of human infections, three leading to death, have already occurred. Existing vaccine strains do not protect against these new viruses, raising a need to identify new vaccine candidate strains. We have developed a novel sequence-based mapping (SBM) tool capable of visualizing complex protein sequence data sets using a single intuitive map. We applied SBM on the complete set of avian H5 viruses in order to better understand hemagglutinin protein variance amongst H5 viruses and identify any patterns associated with this variation. The analysis successfully identified the original reassortments that lead to the emergence of this new subclade of H5 viruses, as well as their known unusual ability to re-assort among neuraminidase subtypes. In addition, our analysis revealed distinct clusters of 2.3.4.4 variants that would not be covered by existing strains in the H5 vaccine stockpile. The results suggest that our method may be useful for pandemic candidate vaccine virus selection.

## Introduction

Highly pathogenic avian influenza viruses (HPAIVs) naturally circulate in migrating aquatic birds resulting in global spread of these viruses[1]. Infected birds can infect poultry flocks leading to massive outbreaks, requiring culling of millions of chickens and other farmed birds. Furthermore, poultry outbreaks put these viruses in direct contact with humans leading to fatal infections in 60% of these cases[2]. Historical avian influenza virus epidemic data is limited. There is, however documentation of a fowl plague, most likely avian influenza, occurring as far back as 1878. Furthermore, more than 18 documented oubreaks have occurred since 1955[3], demonstrating the continuous threat these viruses pose on both humans and birds. The first documented outbreak of the currently circulating H5N1 HPAIV occurred at a goose farm in China in 1996[4]. Since that time, antigenic drift and shift have occurred resulting in new genetically and antigenically distinct lineages and a necessity for new vaccine strains[5]. The first documented H5N1 influenza human infection case was detected in Hong Kong in 1997[4,6,7] and since then avian influenza viruses have been directly infecting humans[2]. This highlights the possibility of these viruses acquiring the ability to transmit efficiently from human to human, leading to a worldwide pandemic. Therefore, there is a global effort to sample these viruses in both avian and mammalian species and develop human vaccines against them in preparation for potential human pandemic.

In 2014 a new variant emerged and spread throughout the globe including, for the first time, North America[8,9]. This new variant, classified as H5NX clade 2.3.4.4[10], combined with multiple neuraminidases (N1, N2, N5, N6, N8) and, after infecting a small number of domestic poultry, led to the culling of at least 40 million chickens or eggs in the US alone[11]. Additionally, four human cases have been reported, one requiring two months of intensive hospital care and the other three resulting in death[5,12].

Emergence of this new highly pathogenic influenza variant, and its rapid spread, reassortment, and the infection of birds in close contact with humans has led to a need for better understanding of these viruses. Additionally, there is very limited antigenic characterization of these viruses[5]. Although two candidate vaccine viruses are in development, whether or not these will protect against all viruses within this clade is unknown.

During these avian outbreaks of antigenically distinct strains it is crucial to be able to test all circulating virus strains for antigenic variants. Unfortunately, time and other resources are often limited during the initial screening process, leading to only a few viruses being antigenically typed. Virus sequence data is often quickly available however and can be used to identify strains that may be antigenically distinct from existing vaccine strains. Moreover, our method can be used to narrow the hundreds of strains isolated during an outbreak into just a few strains requiring antigenic testing increasing the efficiency and quickening the vaccine selection process.

Here we introduce a novel method, SBM, for understanding the variation within the main vaccine target, the hemagglutinin protein. We first apply our method to the available H5 influenza hemagglutinin sequences and investigate patterns of variation among these viruses. We then apply our method to H5 subclade 2.3.4.4 viruses to identify variation patterns and reassortment events. We narrow the 2.3.4.4 subclade strains down to a few clusters of viruses based on the variation in predicted B cell epitopes on the hemagglutinin protein. Lastly, we propose five strains, in addition to the two vaccine candidate strains already in development, that we believe should be antigenically typed further and potentially developed into vaccines.

## Methods

### Influenza Hemagglutinin Protein Sequences

Sequences for the hemagglutinin influenza protein were compiled from the Influenza Resource Database (www.fludb.org) and the World Health Organization’s Global Initiative on Sharing Avian Influenza Data (www.gisaid.org). Protein sequences were filtered down using the following criteria: H5 subtype, HA protein, and complete segments only. This resulted in 4,179 sequences. Sequences were filtered by removing those with missing amino acids, duplications, and those without associated metadata; resulting in 2931 sequences including both avian and human strains. Sequences were aligned using the MUSCLE algorithm and H5 clade classified using the H5 Clade Classification tool (www.fludb.org).

### Sequence-Based Mapping

For each HA sequence, we calculated its hamming distance to all other HA sequences in the data set (see S1 Schematic). The hamming distance is the number of amino acid differences at each residue’s position in the hemagglutinin protein. This results in a distance matrix consisting of the number of amino acid differences between all viruses in the data set. Classical multidimensional scaling (principal coordinates analysis) was performed on the distance matrix using the *stats* package in R. Random noise was added to the epitope specific data plots using the *jitter* function in R in order to visualize overlapping points.

### Visualization of Amino Acid Substitutions on H5 Protein Structure

HA protein structure was created from the A/Vietnam/1203/2004 (H5N1) protein sequence using the automated protein structure homology-modeling server (swissmodel.expasy.org). The number of different amino acids at each position among all 2.3.4.4 clade virus HA sequences was determined and colored on a green-red scale representing the range of amino acids seen at each residue position. Additionally, the number of unique amino acid changes (i.e. the number of different amino acids found at each position of the HA protein among the 2.3.4.4 subclade) was plotted with HA residue position number on the x-axis.

### Phylogenetic Trees

Sequences were aligned using the MUSCLE algorithm and phylogenetic trees were created using the N-J method of the ClustalX 2.0 software. Trees were midpoint rooted, sorted by node order, labeled, and colored using FigTree v1.4.2 software.

## Results

### H5 Hemagglutinin Whole Sequence SBM

**Fig 1:**
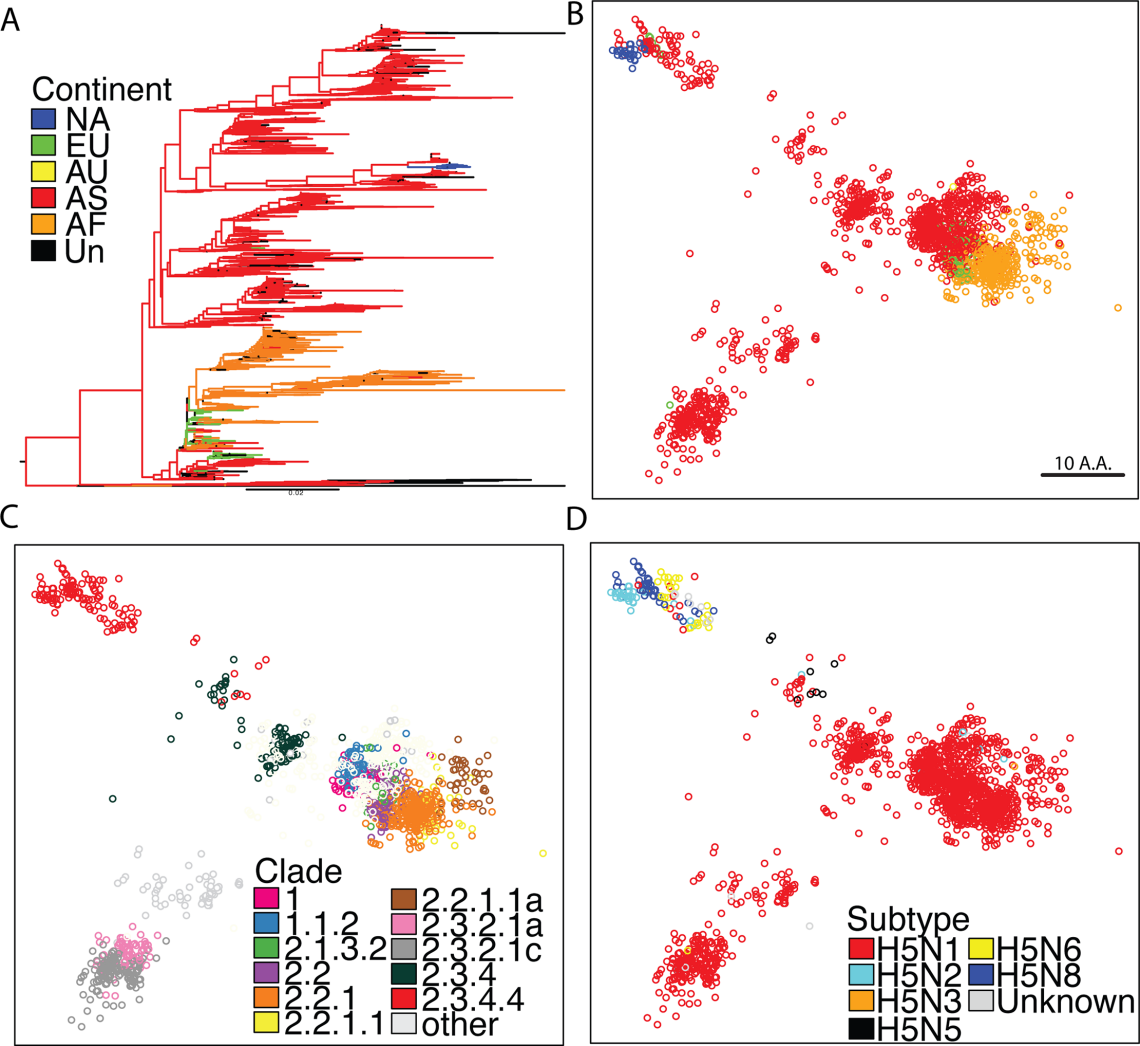
Comprehensive analysis of H5 virus Hemagglutinin. (A) Phylogenetic tree of influenza hemagglutinin proteins constructed from complete set of H5 HA sequences. Nodes were colored by continent of isolation. (B) SBM analysis on all publically available HA sequences for H5 viruses, sequences colored by continent of isolation. (C) Sequences colored by clade, only clades with >50 sequences were colored. (D) Sequences color by NA subtype.

Previous reports have shown that H5 clade 2.3.4.4 viruses are both genetically and antigenically dissimilar to prior circulating H5 viruses[5,13]. Phylogenetic trees were created in order to visualize the lineage associations between H5 HA sequences using all available H5 hemagglutinin complete protein sequences (Fig 1A). Nodes were colored by continent of isolation for each virus strain. North American 2.3.4.4 viruses branch off of Asian isolates as have been reported by others[13]. Although phylogenetic trees are able to capture evolutionary relationships, we were interested in understanding the variation amongst the H5 HA protein and identifying clusters of viruses with similar HAs, which not always easily deciphered using phylogenetic trees. Therefore, we then performed novel analytics that we call sequence-based mapping (SBM; S1 Schematic). This method is based on the theory of shape space in which antigens can be thought of as residing in a space in which the distance between them represents differences in their structure and physiochemical composition (i.e. shape)[14–16]. Our method estimates each HA antigen’s position in shape space by comparing amino acid changes among HA antigen protein sequences. Additionally, we estimate antigenic distance, a measurement of the distance between two antigens in shape space, by only considering changes in predicted B cell epitopes on the HA protein. Both whole sequence and B cell epitope specific distances are used to create two-dimensional maps in order to represent these antigens in shape space.

We first performed SBM on the complete set of H5 viruses mapping variation among the whole HA sequence (Fig 1B–1D). A single large cluster of viruses was found with a smaller cluster of viruses residing adjacent. Additionally, two small clusters are located distal to the large cluster and to each other (Fig 1B–1D). To identify features of this variation, points on the graph were colored by the continent in which the virus was isolated (Fig 1B). The large cluster consists of viruses isolated mostly from Asia and Africa with some Europe and Australia isolated viruses. The smaller cluster located in the bottom-left of the map consists almost exclusively of Asian viruses with a single Europe isolated virus. The cluster residing in the top-left of the map contains the North American stains as well as Asian and European strains which is consistent with previous reports[8]. Interestingly, all clusters contain Asian viruses, indicating the diversity that exists across avian influenza viruses circulating in Asia.

Within the large cluster we see sub-clades 1–2.3.1.1a, but clades 2.3.2.1a and 2.3.2.1c cluster separately demonstrating that although 2.3.1.1a shares a close evolutionary lineage with 2.3.2.1 clades, their HAs are highly distinct. Additionally, clade 2.3.4 forms a unique cluster separate from all other virus clades. Importantly, the top-left virus cluster consists only of clade 2.3.4.4 viruses (Fig 1C). Unlike the phylogenetic analysis (Fig 1A) in which the lineage containing the north American viruses difficult to distinguish from other H5 viruses, our method shows these viruses as a distinct cluster, an important capability when trying to identify emerging novel strains during an avian influenza outbreak.

Next, viruses were labeled according to their neuraminidase (NA) subtype (N1, N2, N3, N5, N6, N8; Fig 1D). The vast majority of the viruses are of the H5N1 2.3.4.4 subtype, although we do find sporadic reassortment within all clusters. Interestingly, H5N5 viruses are phylogenetically classified as clade 2.3.4.4 although they cluster closely to the 2.3.4 virus cluster as well as the 2.3.4.4 virus cluster. This is consistent with initial findings showing the first member of the 2.3.4.4 clade to be a H5N5 virus[17]. Importantly, the unique ability of the 2.3.4.4 clade viruses to undergo reassortment, a trait found in most pandemic viruses, is clearly demonstrated with our method and reveals the method’s ability to incorporate virus metadata to quickly identify high-risk virus strain clusters.

### Variation within the 2.3.4.4 Clade

**Fig 2:**
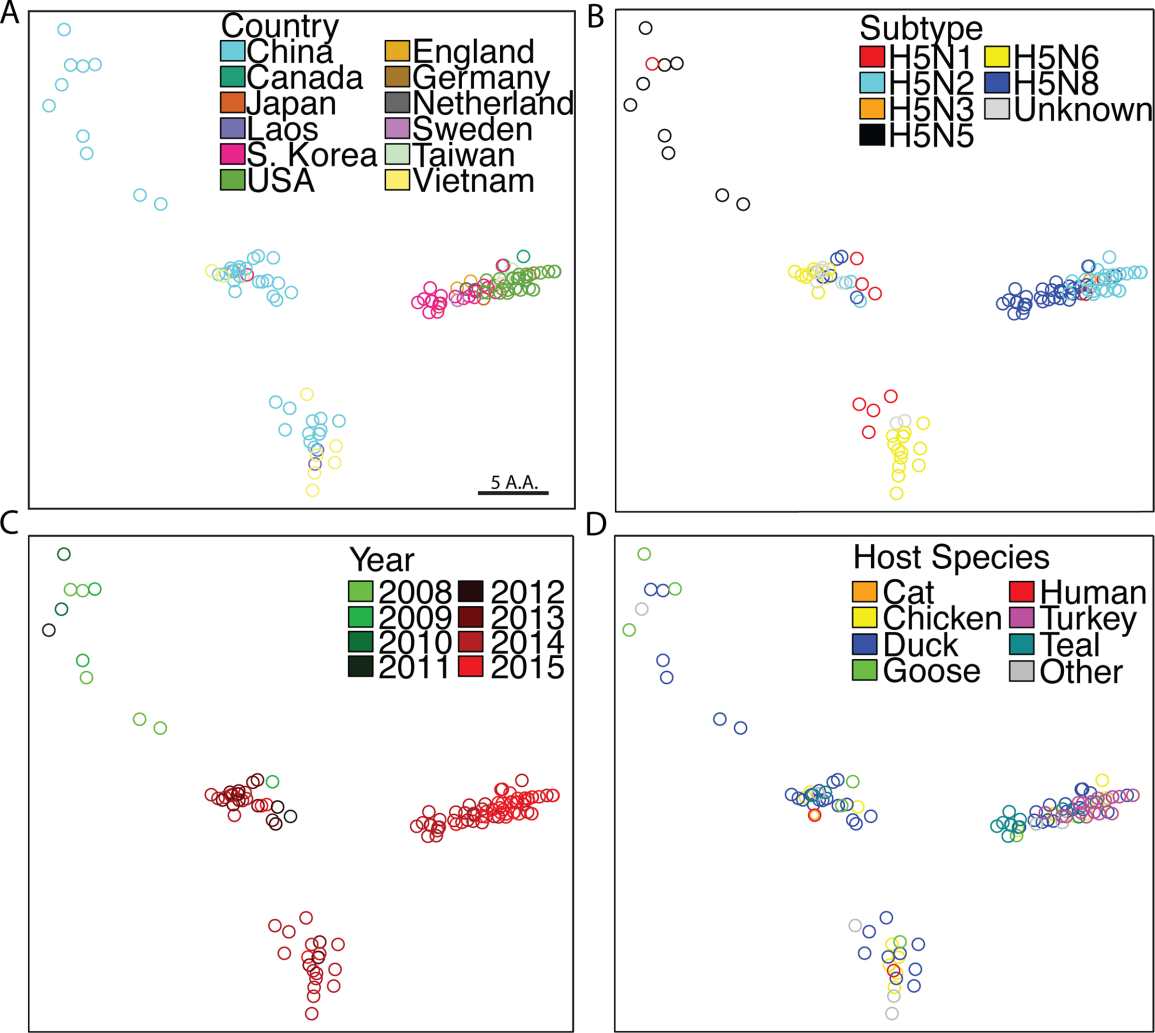
SBM Analysis of clade 2.3.4.4 H5NX viruses. SBM analysis restricted to virus HA sequences belonging to clade 2.3.4.4. (A) HA sequences colored by country of isolation. (B) Sequences colored by NA subtype. (C) Sequences colored by year of isolation as a continuum from green to red (2008–2015 respectively). (D) Sequences colored by species in which virus was isolated.

Given the rapid emergence and dissemination of the 2.3.4.4 clade H5NX viruses, we sought to examine more closely the variance amongst the HA protein of this clade’s viruses. We restricted our protein sequence data set to only those classified as clade 2.3.4.4 viruses and performed SBM (Fig 2A–2D). Multiple clusters amongst the clade were found (Fig 2A). Viruses tended to cluster by geographical region of isolation, with viruses isolated from China residing in multiple clusters. Interestingly, the cluster consisting of viruses isolated from South Korea and Japan cluster separately from viruses insolated in China consistent with reports[18].

Viruses were colored by NA subtype (Fig 2B). H5N5 viruses clustered separately from all other viruses with the exception of H5N1, which appears in almost all clusters. Additionally, the H5N6 viruses appear in two distinct clusters with one cluster containing H5N1, H5N2, H5N6, H5N8 and the other containing only H5N6. Taken together, this demonstrates that within this clade, HA variation clustering does not coincide with NA subtype raising the possibility that multiple reassortment events occurred with each NA subtype during a time of rapid evolution of the HA protein.

To further investigate the evolution of this clade, each point was colored by year of isolation (Fig 2C) or species of isolation (Fig 2D). The H5N5 viruses appear as an almost vertical line starting in 2008, suggesting that H5N5 is the original 2.3.4.4 clade virus, as has been reported[19], and that this virus slowly evolved. Interestingly, the 2008 H5N5 virus resides adjacent to the H5N1 2008 viruses and both were isolated from ducks. The middle and bottom-middle clusters consist mostly of 2011–2012 viruses that were isolated from multiple species including a 2009 H5N1 virus isolated from geese, suggesting that the H5N5 viruses may have reassorted with an H5N1 virus and that this event led to the ability to transmit from ducks to other species, including humans. Within this same clusters we find viruses with multiple NA subtypes, which may also be involved in the spread across species, especially when considering that both the middle and bottom-middle clusters contain human strains indicating these human viruses have distinct HAs but both are of the H5N6 subtype. The right cluster contains the viruses isolated during the most recent outbreak. This cluster contains additional species including teal and turkey. Interestingly, the viruses isolated from teal are the viruses in this cluster most similar to the middle cluster and very similar to goose and chicken viruses isolated in South Korea in this cluster, suggesting that a goose or chicken from either China or Vietnam led to the emergence of this outbreak although it is unclear how these viruses seeded the outbreak in North America.

Taken together, this data suggests that the 2.3.4.4 clade arose in 2008 in China from a H5N1 reassortment with an N5 containing virus in ducks and that these viruses circulated in ducks undergoing another reassortment with H5N1 leading to cross-species transmission and multiple reassortments. At the same time, a distinct HA cluster emerged, possibly from geese, in China and Vietnam. In 2013, a duck containing an H5N8 virus spread the virus to teal in South Korea and eventually to North American poultry.

### Location of Variance on Hemagglutinin

**Fig 3:**
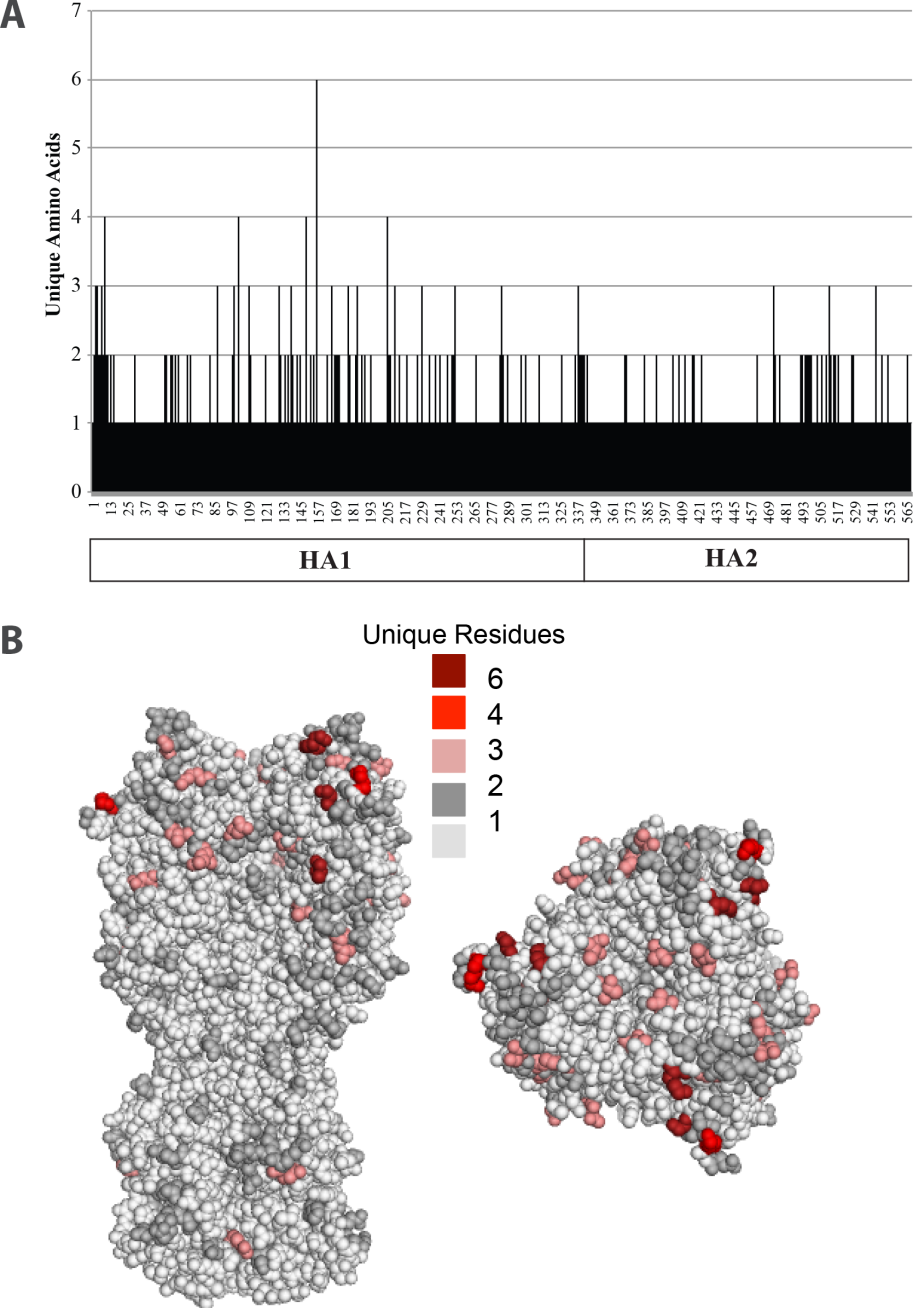
Amino acid substitutions in clade 2.3.4.4 H5NX viruses. (A) Number of unique amino acids found at each position (1–568) across H5NX viruses. HA1 and HA2 regions of the hemagglutinin protein are indicated in box. (B) Mapping of unique amino acids onto 3D reconstruction of H5N1 HA. Number of unique amino acids found at each amino acid position is colored as depicted in legend.

Although mutations in influenza viruses occur predominantly in the head region, it is not known where mutations occur amongst the H5 2.3.4.4 clade. Understanding where this variation occurs is important since amino acid changes in the head region of HA have been shown to produce viruses able to evade neutralizing antibodies induced by vaccination[20]. The number of unique amino acids found at each amino acid position were calculated across the HA protein (Fig 3A). Most of amino acid variability was found in the HA1 domain with a substantial number of positions contained more then one amino acid in both regions. The HA1 region had 88 positions with at least two amino acids and HA2 35 positions. 21 positions are highly variable with 3–6 amino acids found at those positions. Interestingly, viruses with changes in positions 143 and 145 were found; single mutations in either of these positions have been shown to allow escape from antibody mediated neutralization[21]. Moreover, we found that amino acid 156 contains 6 different amino acids at this position, including K and E amino acids. Specifically the K156E substitution, has been shown to allow escape from antibody neutralization. Together, amino acid substitutions amongst the 2.3.4.4 clade are consistent with antigenic variation within the clade.

**Fig 4:**
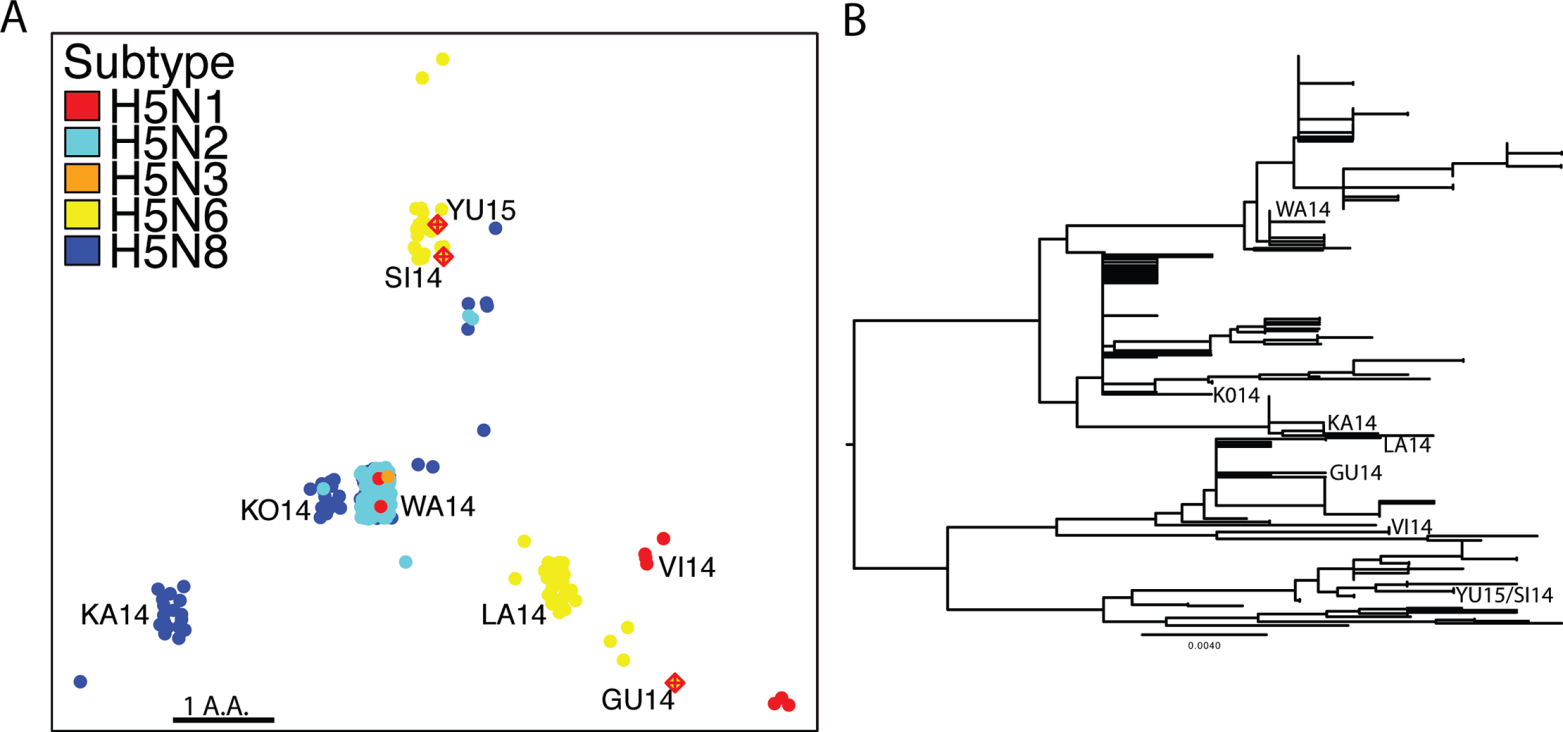
Variation within the B cell epitopes of recent clade 2.3.4.4 H5NX viruses. **(A)** SBM was performed on truncated sequences contain only amino acids within B cell epitopes. Sequences colored by subtype. Crossed-diamond symbols indicate human infection sequences. (B) Phylogenetic tree of 2.3.4.4 subclade HA. Labels were added for comparison with Fig 4A.

Substitutions were mapped onto a 3 dimensional reconstruction of the A/Vietnam/-1203/2004 (H5N1, Clade 1). A considerable amount of variation around the receptor-binding domain was found (Fig 4B), which may further indicate that antigenically distinct variants exist amongst this clade[21]. Although epitopes specific to the 2.3.4.4 subclade have not been identified, it has been reported that H5N1 epitopes reside within regions corresponding to the A and B epitopes of H3N2 viruses and the Sa epitopes of H1N1 viruses[22]. Under the assumption that these epitopes are similar in the emerging subclade, we analyzed the amino acid differences within the areas predicted to be epitopes based on the residues residing in epitopes of the H5N1 strain. Surprisingly, 70 amino acid differences were found within these epitopes amongst the clade. Taken together, this data indicates there is considerable variation in regions of the H5 hemagglutinin known to bind neutralizing antibodies.

### B cell Epitope SBM

Given the multiple human infections that have occurred among the 2.3.4.4 clade and the amino acid variability found in the H1 domain across these viruses, we attempted to identify potential antigenic variants within the 2.3.4.4 clade. We performed a variant of SBM where only amino acid variation is the predicted H5N1 epitopes[23] is used for mapping. Additionally, only HA sequences from viruses isolated since 2013 where used, since these are the viruses most recently circulating.

The viruses generally fall into 4 regions on the map, those on the top, middle, bottom-left, and bottom-right. The four human infections fall into two clusters, one containing A/Sichuan/26221/2014 (SI14), A/Yunnan/0127/2015 and A/Yunnan/14563/2015 (YU15) in the top region and the other containing A/Guangzhou/39715/2014 (GU14) in the bottom-right region. The top region contains two human infection H5N6 strains as well as H5N8 and H5N2 strains. The middle region contains all subtypes but H5N6 and two distinct groups of viruses exist in this region, the WA14 viruses as well as A/broiler duck/Korea/H48/2014 (KO14) viruses. The bottom-left region contains a single group of H5N8 viruses including A/mallard duck/Kagoshima/KU116/2015 (KA14). The bottom-left region contains mostly H5N6 viruses, including the human infection strain A/Guangzhou/39715/2014 (GU14), as well as H5N1 strains. Importantly, there are viruses distinct from the human infecting strain GU14, including another H5N6 strain A/chicken/Laos/LPQ001/2014 (LA14) and the H5N1 strain A/duck/Vietnam/LBM632/2014 (VI14).

The amino acid variation within the predicted epitopes of this clade occurred in 9 different residue positions out of the 45 epitope-residing amino acids (Table 1). Residue 156 had the most variability with 3 different amino acids found at this position. Although the contribution to antigenic difference cannot be determined by our method, even a single amino acid substitution has been shown to cause an antigenic change between viruses[21]. Moreover, these seven clusters are not readily identified with phylogenetic analysis (Fig 4B) demonstrating SBM uniqueness.

**Table 1:** Epitope Amino Acid Differences Contributing to PC1 and PC2 position for 2.3.4.4. H5NX Strains.

Epitope	A	A	A	B	B	B	B	Sa	Sa
Residue Position	140	156	160	172	179	205	214	177	178
SI14	N	T	F	A	S	N	I	K	I
YU15	N	T	F	A	S	N	I	K	I
WA14	N	A	F	A	S	N	V	K	I
GU14	N	M	F	T	S	N	V	K	M
VI14	D	M	F	T	S	E	V	K	I
KO14	N	A	F	A	N	N	V	K	I
LA14	N	M	F	A	S	N	V	K	M
KA15	N	A	Y	A	S	N	V	E	I

Two viruses have been developed as vaccines, A/gyrfalcon/Washington/41088-6/2014 (WA14) and A/Sichuan/26221/2014 (SI14). It has been previously shown that antiserum from SI14 infected ferrets has low titers against A/gyrfalcon/Washington/41088-6/2014 (WA14)[5], consistent with their distinct locations on our SBM map. Our analysis suggests that the GUI4 human virus may also react poorly with SI14 antiserum and should be included in vaccine efficacy testing. Additionally, we find four other clusters of viruses A/broiler duck/Korea/H48/2014 (KO14), A/duck/Vietnam/LBM632/2014 (VI14), A/mallard duck/Kagoshima/KU116/2015 (KA14) and A/chicken/Laos/LPQ001/2014 (LA14) that may also be antigenically distinct from these vaccine strains. Importantly, a virus similar to LA14 has been shown to have reduced titers to SI14 antiserum and little cross-reactivity to WA14 antiserum[24] as well as being of the H5N6 subtype, the only subtype in this clade that has infected humans.

### Candidate Vaccine Viruses

Based on our analysis we suggest seven viruses to be included in the pandemic preparedness candidate vaccine viruses selection process performed by the World Health Organization[25], one from each cluster in Fig 4: A/Sichuan/26221/2014, A/Guangzhou/39715/2014, A/gyrfalcon/Washington/41088-6/2014, A/broiler duck/Korea/H48/2014, A/duck/Vietnam/LBM632/2014, A/mallard duck/Kagoshima/KU116/2015, and A/chicken/Laos/LPQ001/2014. A list of viruses in each Fig 4 cluster has been included to provide possible alternatives (S1 Table).

Overall, we have found that the hemagglutinin proteins from 2.3.4.4 are distinct from other H5 viruses and find considerable variability within the 2.3.4.4 clade exists. Evaluation of regions known to bind neutralizing antibodies demonstrates potential antigenically distinct viruses. Last, our analysis suggests inclusion of seven viruses in H5NX clade 2.3.4.4 candidate vaccine efficacy testing.

## Discussion

Since the discovery of the currently circulating H5N1 highly pathogenic avian influenza viruses, these H5N1 viruses have steadily evolved resulting in ten phylogenetic clades and 43 sub clades. Additionally, a new genetic clade containing the H5 gene (2.3.4.4) emerged and subsequently spread from Asia to Europe and North America[8]. Here we introduced a novel method for visualizing hemagglutinin protein variance.

Inspiration for our method stems from the fundamentals of Shape Space theory (S1 Schematic)[14]. In the theory, antigen-antibody complementary binding regions are thought of as points existing in a defined space and the distance between these points translates to differences in the shape of the antibody-antigen binding region. This distance is known as the antigenic distance and has been used to understand antigenic differences between viruses. The first attempt to visualize viruses in shape space came in 2004 with the introduction of antigenic cartography by Smith et al. (2004). This method placed viruses on a graph using ferret antiserum and hemagglutinin inhibition assay (HI) titers to determine antigenic distance.

Since 2004, many methods have been developed that use hemagglutinin protein sequences to estimate antigenic distance[26–29]. These studies have demonstrated that hemagglutinin sequence information can be used to accurately estimate antigenic distance with even greater accuracy when B cell epitopes are known[30,31]. All attempts to use sequence data to calculate antigenic distance have been performed using seasonal viruses and no attempts have been made to use these methods to identify potential pandemic avian viruses.

Although immune assays and analytical tools such as antigenic cartography are needed to fully characterize an influenza virus, these methods require virus culture, production of antiserum in experimental animals, and performing HI assays, methods which together require substantial labor, time, and funding[32]. Additionally, some viruses have been found that do not bind to red blood cells[33], a requirement for HI assays, while other viruses acquire mutations during *in vivo* growth leading to antigenic variation not seen in the infecting viruses[34,35]. Additionally, restrictions are underway to limit use and distribution of HPAIV and thus immunological assays will be restricted to labs with permission and the appropriate (and expensive) BSL3+ facilities. Alternatively, direct viral RNA sequencing from nasopharyngeal swabs is inexpensive, timely, and publically available. Application of sequence-based antigenic distance calculations and subsequent visualization of avian viruses can circumvent these challenges and lead to quick and novel insights into emerging viruses.

Our analysis provides a different perspective on H5 viruses compared to traditional phylogenetic analysis. Phylogenetic analysis displays sequence relationships using connecting lines where the sequence similarity is proportional to the length of the line. Phylogenetic analysis of the sequences used in this study are difficult interpret (Fig 1A), particularly for non-experts in the method, and patterns are not as intuitive. Our method places the sequences as points in space, where the distance between any two points represents the extent of sequence difference. Therefore, we believe our method provides a more intuitive way to understand differences, especially when performing comprehensive analysis.

The unusual ability to recombine with many NA genes seems to be distinct to this clade, although it is not clear if this property arose in the H5N1 virus prior to reassortment with N5 or if this property arose in the evolving H5N5 virus. Phylogenetic analysis has demonstrated H5N5 viruses emerged first, but their relationship to the rest of the 2.3.4.4 clade is difficult to decipher[5,9]. It has been suggested that multiple reassortment events between H5N1 and other viruses lead to the emergence of multiple subtypes[8]. Our analysis suggests that H5N5 viruses emerged first and then evolved over time eventually giving rise to other 2.3.4.4 viruses. Importantly, the increased reassortments in this clade, regardless of ancestry, raise the potential danger of these viruses to the human population. Reassortment between viruses has lead to all major pandemics in the 20^th^ and 21^st^ centuries[36,37].

One interesting result of our analysis is how clade nomenclature can be misleading. Phylogenetic analysis aims to determine taxa and identify an ancestor and all its descendants. Therefore clades are named in accordance with which ancestor gave rise to that particular clade. Our analysis demonstrates that the nomenclature can obscure important relationships. For instance, clade 2.3.4 sequences are more similar to clade 1 viruses than 2.3.2.1 or 2.3.4.4 in our analysis, though nomenclature would suggest differently. Therefore, even though both SBM analysis and phylogenetic analysis suggest 2.3.4 viruses were the ancestors of 2.3.4.4 viruses, our method suggests that overall the 2.3.4 clade is much closer to clade 1 viruses than 2.3.4.4 viruses.

Our antigenic analysis focused on B cell epitope differences as well as full sequence differences. Although neutralization antibodies primarily bind 3D structures of the known epitopes, T cell epitopes exist as linear peptides that can reside anywhere along the protein sequence. Recently, it has been demonstrated that T cells can be limiting during influenza infectious[38]. Therefore, our analysis may also be used to determine divergent T cell epitopes. Fig 2 demonstrates the diversity across the 2.3.4.4 clade hemagglutinin, which may indicate a failure to elicit cross-reactive T cells with a single vaccine strain from this clade—information not determined from HI assays.

Although sequence-based methods have been shown to correlate well with antigenic diversity, there are limitations to the approach. Firstly, it has been shown that glycosylation of the hemagglutinin protein can lead to antigenically distinct viruses[39] and this is not captured by our methods. Additionally, we treated all amino acid substitutions equally without regards to the specific amino acids. Each amino acid has distinct properties[40], and these differences may need to be taken into account to fully calculate antigenic differences. Lastly, we used primary sequence structure when calculating differences ignoring secondary and tertiary structure. Although these differences have not been taken into account in the current method, methods have been developed that allow incorporation of these data[41], providing a path for further refinement of the tool.

Four human infections have occurred since the emergence of the 2.3.4.4 clade. Three of these A/Sichuan/26221/2014, A/Yunnan/14563/2015, and A/Yunnan/0127/2015 have identical B cell epitope protein sequences and A/Sichuan/26221/2014 is currently in development as a vaccine[5,13,25]. The other human virus, A/Guangzhou/39715/2014, is genetically distinct from the A/Sichuan/26221/2014 and differs in amino acids located in B cell epitopes. This suggests that there may be two antigenically distinct, human infecting, viruses. Our method found North American virus epitopes are also distinct from A/Sichuan/26221/2014 in agreement with initial characterization using HI assays showing low cross reactive titers between the viruses[5]. Furthermore, the World Health Organization has only suggested two vaccines strains A/Sichuan/26221/2014 (H5N6) and A/gyrfalcon/Washington/41088-6/2014 (H5N8) to protect against the 2.3.4.4 clade. Our analysis clearly demonstrates that other viruses contain distinct B cell epitopes suggesting that there may be viruses antigenically distinct from these two strains within the 2.3.4.4 clade. We propose that these viruses be further characterized antigenically by the World Health Organization to determine if additional vaccine strains are needed to fully protect against the 2.3.4.4 clade.

## Supporting Information

S1 Schematic Visual Representation of SBM method. Three representative 2.3.4.4 viruses (SI14, WA14, GU14) shortened to only amino acids residing in B cell epitope amino acids that differed between any hemagglutinin sequences for viruses in this clade (Table 1). Shape differences are estimated by comparing changes in amino acids at each position of the hemagglutinin protein. Sequences are compared pairwise and amino acid differences are determined by hamming distance (the number of amino acids that differ between the strains). Second, a distance matrix is compiled from these calculations. Classical (metric) multidimensional scaling (principal coordinates analysis) is performed on the distance matrix in order to reduce dimensions (2D) but preserve distances allowing the distance matrix to be visualized. Last, a scale bar is included representing the number of amino acids differences between two sequences at that distance.
(TIFF)

S1 Table H5 2.3.4.4 Clade Sequences. Strain names for each H5 2.3.4.4 residing in the seven clusters (Fig 4).
(DOCX)
