# Supplementary figures and images for: Novel Sequence-Based Mapping of Recently Emerging H5NX Influenza Viruses Reveals Pandemic Vaccine Candidates
Source: PLoS One. 2016 Aug 5;11(8):e0160510. doi: 10.1371/journal.pone.0160510 (PMC4975393; doi:10.1371/journal.pone.0160510)

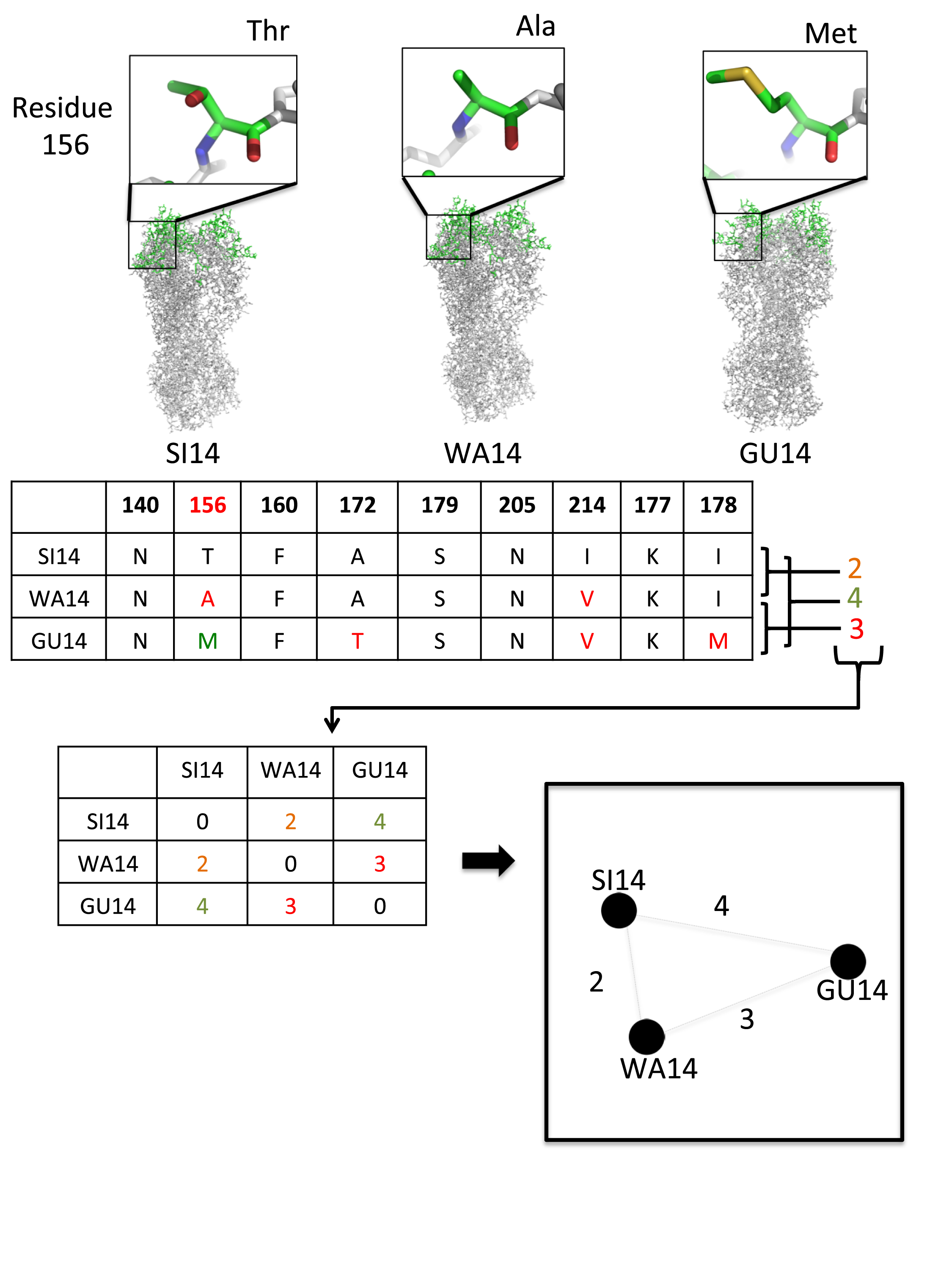

Supplement: S1 Schematic — Three representative 2.3.4.4 viruses (SI14, WA14, GU14) shortened to only amino acids residing in B cell epitope amino acids that differed between any hemagglutinin sequences for viruses in this clade (Table 1). Shape differences are estimated by comparing changes in amino acids at each position of the hemagglutinin protein. Sequences are compared pairwise and amino acid differences are determined by hamming distance (the number of amino acids that differ between the strains). Second, a distance matrix is compiled from these calculations. Classical (metric) multidimensional scaling (principal coordinates analysis) is performed on the distance matrix in order to reduce dimensions (2D) but preserve distances allowing the distance matrix to be visualized. Last, a scale bar is included representing the number of amino acids differences between two sequences at that distance. (TIFF) [file pone.0160510.s001.tiff]
