# Supplementary material for: Novel Sequence-Based Mapping of Recently Emerging H5NX Influenza Viruses Reveals Pandemic Vaccine Candidates
Source: PLoS One. 2016 Aug 5;11(8):e0160510. doi: 10.1371/journal.pone.0160510 (PMC4975393; doi:10.1371/journal.pone.0160510)
Supplement: S1 Table — Strain names for each H5 2.3.4.4 residing in the seven clusters (Fig 4). (DOCX) [file pone.0160510.s002.docx]

| **WA14 Cluster** |
| --- |
| A/pheasant/Washington/3147-2/2015 |
| A/chicken/Wisconsin/15-012160-1/2015 |
| A/chicken/Nebraska/15-017990-5/2015 |
| A/Northernpintail/Washington/40964/2014 |
| A/chicken/Washington/3490-18/2015 |
| A/mallard/Korea/W452/2014 |
| A/turkey/MN/10915-1/2015 |
| A/crane/Kagoshima/KU1/2014 |
| A/chicken/Iowa/14399-4/2015 |
| A/chicken/Netherlands/14015531/2014 |
| A/duck/England/36038/14 |
| A/duck/Chiba/26-372-61/2014 |
| A/broiler duck/Korea/Buan2/2014 |
| A/chicken/BC/FAV9/2014 |
| A/Baikal teal/Korea/H68/2014 |
| A/baikal teal/Korea/1437/2014 |
| A/turkey/Iowa/13541-1/2015 |
| A/chicken/Washington/61-9/2014 |
| A/breeder chicken/Korea/H250/2014 |
| A/chicken/Oregon/41613-2/2014 |
| A/chicken/Nebraska/15-017897-1/2015 |
| A/baikal teal/Korea/1441/2014 |
| A/chicken/Iowa/14399-4/2015 |
| A/chicken/Kansas/8395-3/2015 |
| A/duck/Netherlands/14015898/2014 |
| A/turkey/Iowa/11762-1/2015 |
| A/turkey/Wisconsin/15-012012-2/2015 |
| A/turkey/ND/11419-1/2015 |
| A/pheasant/Washington/3147-2/2015 |
| A/white-fronted goose/Korea/H231/2014 |
| A/chicken/Taiwan/a174/2015 |
| A/waterfowl/Korea/S005/2014 |
| A/domesticduck/Washington/61-16/2014 |
| A/turkey/MN/11668-1/2015 |
| A/broiler duck/Korea/H133/2014 |
| A/Baikal teal/Korea/H96/2014 |
| A/duck/England/36254/14 |
| A/gyrfalcon/Washington/41088-6/2014 |
| A/turkey/Minnesota/9892-2/2015 |
| A/chicken/Kansas/8395-3/2015 |
| A/chicken/BC/FAV24/2014 |
| A/turkey/BC/FAV10/2014 |
| A/baikal teal/Korea/2402/2014 |
| A/bean goose/Korea/H328/2014 |
| A/turkey/Minnesota/9892-2/2015 |
| A/Chicken/Netherlands/14015526/2014 |
| A/turkey/Minnesota/9845-4/2015 |
| A/chicken/Montana/15-010559-1/2015 |
| A/chicken/Wisconsin/15-011595-1/2015 |
| A/Baikal teal/Korea/H80/2014 |
| A/turkey/BC/FAV10/2014 |
| A/turkey/NorthDakota/15-013049-1/2015 |
| A/environment/Kagoshima/KU-ngr-H/2014 |
| A/poultry/BC/FAV19/2014 |
| A/turkey/Iowa/14319-1/2015 |
| A/chicken/Iowa/14322-6/2015 |
| A/baikal teal/Korea/Donglim3/2014 |
| A/turkey/California/K1500169-1.2/2015 |
| A/chicken/BC/FAV9/2014 |
| A/goose/Taiwan/a015/2015 |
| 540667_A/mallard_duck/Korea/W452/2014 |
| A/domestic duck/Washington/61-16/2014 |
| A/turkey/Iowa/11762-1/2015 |
| A/chicken/Oregon/A01819044/2015 |
| A/crane/Kagoshima/KU13/2014(H5N8) |
| A/poultry/BC/FAV15/2014 |
| A/guineafowl/Oregon/41613-1/2014 |
| A/baikal teal/Korea/1456/2014 |
| A/chicken/Iowa/04-20/2015 |
| A/gyrfalcon/Washington/41088-6/2014 |
| A/mallard/Korea/H1924-6/2014 |
| A/turkey/Iowa/14318-1/2015 |
| A/chicken/BC/FAV8/2014 |
| A/Americangreen-wingedteal/Washington/195750/2014 |
| A/chicken/Iowa/13542-2/2015 |
| A/chicken/Washington/61-9/2014 |
| A/turkey/SouthDakota/15-010371/2015 |
| A/turkey/Washington/61-22/2014 |
| A/turkey/Missouri/7458-1/2015 |
| A/Chicken/Netherlands/14015824/2014 |
| A/turkey/Arkansas/7791-1/2015 |
| A/Baikal teal/Korea/H62/2014 |
| A/turkey/Minnesota/9845-4/2015 |
| A/turkey/Washington/61-22/2014 |
| A/common teal/Korea/H455-30/2014 |
| A/turkey/Minnesota/7172-1/2015 |
| A/chicken/Netherlands/14016437/2014 |
| A/turkey/Missouri/7458-1/2015 |
| A/baikal teal/Korea/1449/2014 |
| A/breeder duck/Korea/H158/2014 |
| A/baikal teal/Korea/2416/2014 |
| A/turkey/California/K1500169-1.2/2015 |
| A/poultry/BC/FAV17/2014 |
| A/chicken/Netherlands/14015766/2014 |
| A/chicken/Iowa/14589-1/2015 |
| A/turkey/NorthDakota/15-011420-13/2015 |
| A/chicken/Washington/3490-18/2015 |
| A/broiler duck/Korea/H32/2014 |
| A/chicken/kumamoto/1-7/2014(H5N8) |
| A/duck/Taiwan/a068/2015 |
| A/chicken/Iowa/14589-1/2015 |
| A/turkey/Iowa/14318-1/2015 |
| 540673_A/Gyrfalcon/Washington/41088-6/2014 |
| A/chicken/Oregon/A01819044/2015 |
| A/duck/Chiba/26-372-48/2014 |
| A/turkey/BC/FAV10/2014 |
| A/breeder duck/Korea/H200/2014 |
| A/breeder chicken/Korea/H122/2014 |
| A/turkey/Arkansas/7791-1/2015 |
| A/turkey/Iowa/13541-1/2015 |
| A/chicken/BC/FAV21/2014 |
| A/chicken/BC/FAV20/2014 |
| A/turkey/SD/11089-3/2015 |
| A/chicken/Minnesota/15-013533-1/2015 |
| A/duck/Taiwan/a043/2015 |
| A/tundra swan/Korea/H411/2014 |
| A/turkey/MN/10777/2015 |
| A/chicken/BC/FAV8/2014 |
| A/baikal teal/Korea/1446/2014 |
| A/turkey/Minnesota/7172-1/2015 |
| A/American green-winged teal/Washington/195750/2014 |
| A/duck/England/36226/14 |
| A/broiler duck/Korea/H65/2014 |
| 540672_A/Northern_Pintail/Washington/40964/2014 |
| A/Baikal teal/Korea/H84/2014 |
| A/baikal teal/Korea/K14-E016/2014 |
| A/turkey/Germany/AR2485-86-L00899/2014 |
| A/turkey/Iowa/14319-1/2015 |
| A/Baikal teal/Korea/H41/2014 |
| A/chicken/Iowa/14322-6/2015 |
| A/turkey/BC/FAV14/2014 |
| A/chicken/Iowa/13542-2/2015 |
| A/spot-billed duck/Korea/H455-42/2014 |
| A/mallard/Korea/H297/2014 |
| A/Northern pintail/Washington/40964/2014 |
| **KO14 Cluster** |
| A/Koreannativechicken/Korea/H1847/2014 |
| A/broiler duck/Korea/H49/2014 |
| A/broiler duck/Korea/H48/2014 |
| A/broilerduck/Korea/H1803/2014 |
| A/baikal teal/Korea/1457/2014 |
| A/breederduck/Korea/H1752/2014 |
| A/broilerduck/Korea/H1755/2014 |
| A/Koreannativechicken/Korea/H1903/2014 |
| A/baikal teal/Korea/1445/2014 |
| A/broilerduck/Korea/H1840/2014 |
| A/broilerduck/Korea/H1763/2014 |
| A/bean goose/Korea/H40/2014 |
| A/broilerduck/Korea/H1864/2014 |
| A/broilerduck/Korea/H1839/2014 |
| A/baikal teal/Korea/1447/2014 |
| A/baikal teal/Korea/1458/2014 |
| A/chicken/BC/FAV23/2014 |
| **SI14 Cluster** |
| A/black chicken/Jiangxi/10139/2014(mixed) |
| A/chicken/Zhejiang/6C2/2013 |
| A/duck/Vietnam/1434/2014 |
| A/duck/Vietnam/1144/2014 |
| A/environment/Sichuan/NCLL1/2014 |
| A/cat/Sichuan/SC18/2014 |
| A/Anas_crecca/Hubei/Chenhu1623-5/2014 |
| A/Sichuan/26221/2014 |
| A/black chicken/Jiangxi/10129/2014(H5N6) |
| A/chicken/Shenzhen/1845/2013 |
| A/Yunnan/0127/2015 |
| A/duck/Vietnam/1152/2014 |
| A/duck/Vietnam/1507/2014 |
| A/chicken/Sichuan/NCJPL1/2014 |
| A/duck/Sichuan/NCXJ16/2014 |
| A/duck/Vietnam/HU1-1152/2014 |
| A/duck/Sichuan/NCJPL7/2014 |
| A/environment/Zhenjiang/C13/2013 |
| A/duck/Jiangxi/13469/2014 |
| A/duck/Sichuan/NCXJ15/2014 |
| A/swan goose/Jilin/JL01/2014 |
| A/muscovy duck/Quang Ninh/5c112/2013 |
| A/duck/Jiangxi/13475/2014 |
| A/duck/Vietnam/HU1-1144/2014 |
| A/chicken/Shenzhen/712/2013 |
| A/black chicken/Jiangxi/10131/2014(mixed) |
| A/mallard/Shanghai/SH-9/2013 |
| A/duck/Zhejiang/W24/2013 |
| A/duck/Zhejiang/6D18/2013 |
| A/breeder duck/Korea/Gochang1/2014 |
| A/duck/Eastern China/1111/2011 |
| A/goose/Eastern China/1112/2011 |
| A/duck/Ningbo/3262/2013 |
| A/duck/Shandong/Q1/2013 |
| **KA14 Cluster** |
| A/Koreannativechicken/Korea/H1747/2014 |
| A/crane/Kagoshima/KU21/2014(H5N8) |
| A/crane/Kagoshima/KU53/2015(H5N8) |
| A/commonteal/Korea/KU-12/2015 |
| A/mallardduck/Kagoshima/KU70/2015(H5N8) |
| A/greaterwhite-frontedgoose/Korea/K14-372-2/2014 |
| A/chicken/Miyazaki/7/2014 |
| A/mallard/Korea/H2003/2014 |
| A/greaterwhite-frontedgoose/Korea/K14-369-3/2014 |
| A/mallard/Korea/KU3-2/2015 |
| A/mallard/Korea/H1991/2014 |
| A/spot-billedduck/Korea/H1981/2014 |
| A/crane/Kagoshima/KU41/2014(H5N8) |
| A/greaterwhite-frontedgoose/Korea/K14-367-4/2014 |
| A/greaterwhite-frontedgoose/Korea/K14-371-4/2014 |
| A/mandarinduck/Korea/K14-366-1/2014 |
| A/mallardduck/Kagoshima/KU116/2015(H5N8) |
| A/mandarinduck/Korea/K14-367-1/2014 |
| A/greaterwhite-frontedgoose/Korea/K14-374-1/2014 |
| **LA14 Cluster** |
| A/chicken/Dongguan/3363/2013(H5N6) |
| A/chicken/Shenzhen/1395/2013 |
| A/duck/Vietnam/LBM759/2014 |
| A/chicken/Dongguan/1100/2014(mixed) |
| A/chicken/Shenzhen/433/2013 |
| A/muscovyduck/Vietnam/LBM755/2014 |
| A/silkie chicken/Dongguan/2809/2013(H5N6) |
| A/chicken/Shenzhen/552/2013 |
| A/chicken/Jiangxi/NCDZT1126/2014 |
| A/muscovyduck/Vietnam/LBM754/2014 |
| A/duck/Vietnam/LBM751/2014 |
| A/chicken/Dongguan/2690/2013(H5N6) |
| A/muscovyduck/Vietnam/LBM757/2014 |
| A/duck/Vietnam/LBM760/2014 |
| A/goose/Shantou/1763/2014 |
| A/chicken/Laos/LPQ001/2014 |
| A/duck/Vietnam/LBM759/2014 |
| A/chicken/Shenzhen/2396/2013 |
| A/muscovy duck/Vietnam/LBM755/2014 |
| A/chicken/Shenzhen/2269/2013 |
| A/chicken/Shenzhen/1061/2013 |
| A/muscovyduck/Vietnam/LBM756/2014 |
| A/chicken/Jiangxi/NCDZT1123/2014 |
| A/duck/Vietnam/LBM758/2014 |
| A/Black-crowned night heron/Vietnam/WBT198/2014 |
| A/duck/Vietnam/LBM752/2014 |
| A/duck/Dongguan/3069/2013 |
| A/duck/Vietnam/LBM752/2014 |
| A/duck/Vietnam/LBM751/2014 |
| A/duck/Vietnam/LBM760/2014 |
| A/Chinese pond heron/Vietnam/WBT231/2014 |
| A/chicken/Laos/XBY003/2014 |
| A/duck/Vietnam/LBM758/2014 |
| A/duck/Dongguan/2685/2013 |
| A/chicken/Shenzhen/2072/2013 |
| **GU14 Cluster** |
| A/duck/Guangdong/GD01/2014 |
| A/chicken/Dongguan/4259/2013(H5N6) |
| A/Common moorhen/Vietnam/WBT226/2014 |
| A/Guangzhou/39715/2014 |
| **VI14 Cluster** |
| A/duck/Vietnam/LBM638/2014 |
| A/muscovy duck/Vietnam/LBM635/2014 |
| A/duck/Vietnam/LBM632/2014 |
| A/muscovy duck/Vietnam/LBM636/2014 |
